# Esophageal Squamous Cell Carcinoma Is Accompanied by Local and Systemic Changes in L-arginine/NO Pathway

**DOI:** 10.3390/ijms21176282

**Published:** 2020-08-30

**Authors:** Iwona Bednarz-Misa, Paulina Fortuna, Mariusz G. Fleszar, Łukasz Lewandowski, Dorota Diakowska, Joanna Rosińczuk, Małgorzata Krzystek-Korpacka

**Affiliations:** 1 Department of Medical Biochemistry, Wroclaw Medical University, 50-368 Wroclaw, Poland; iwona.bednarz-misa@umed.wroc.pl (I.B.-M.); paulina.fortuna@umed.wroc.pl (P.F.); fleszar.mariusz@gmail.com (M.G.F.); lukasz.lewandowski@umed.wroc.pl (Ł.L.); 2 Department of Gastrointestinal and General Surgery, Wroclaw Medical University, 50-368 Wroclaw, Poland; dorota.diakowska@umed.wroc.pl; 3 Department of Nervous System Diseases, Wroclaw Medical University, 51-618 Wroclaw, Poland; joanna.rosinczuk@umed.wroc.pl

**Keywords:** asymmetric dimethylarginine (ADMA), symmetric dimethylarginine (SDMA), ornithine, citrulline, dimethylamine (DMA), arginase (ARG), nitric oxide synthase (NOS), protein arginine N-methyltransferase (PRMT), dimethylarginine dimethylaminohydrolase (DDAH), ornithine decarboxylase (ODC)

## Abstract

The L-arginine/NO pathway holds promise as a source of potential therapy target and biomarker; yet, its status and utility in esophageal squamous cell carcinoma (ESCC) is unclear. We aimed at quantifying pathway metabolites in sera from patients with ESCC (*n* = 61) and benign conditions (*n* = 62) using LC-QTOF-MS and enzyme expression in esophageal tumors and matched noncancerous samples (*n* = 40) using real-time PCR with reference to ESCC pathology and circulating immune/inflammatory mediators, quantified using Luminex xMAP technology. ESCC was associated with elevated systemic arginine and asymmetric dimethylarginine. Citrulline decreased and arginine bioavailability increased along with increasing ESCC advancement. Compared to adjacent tissue, tumors overexpressed *ODC1*, *NOS2*, *PRMT1*, and *PRMT5* but had downregulated *ARG1*, *ARG2*, and *DDAH1*. Except for markedly higher *NOS2* and lower *ODC1* in tumors from M1 patients, the pathology-associated changes in enzyme expression were subtle and present also in noncancerous tissue. Both the local enzyme expression level and systemic metabolite concentration were related to circulating inflammatory and immune mediators, particularly those associated with eosinophils and those promoting viability and self-renewal of cancer stem cells. Metabolic reprogramming in ESCC manifests itself by the altered L-arginine/NO pathway. Upregulation of *PRMT*s in addition to *NOS2* and *ODC1* and the pathway link with stemness-promoting cytokines warrants further investigation.

## 1. Introduction

Squamous cell carcinoma of the esophagus (ESCC) is the dominant type of esophageal cancerand one of the deadliest and understudied malignancies worldwide. The etiology of ESCC differs by geographic region with smoking and heavy alcohol consumption considered synergistic primary causes in Western European countries. New potential factors, such as polycyclic aromatic hydrocarbons; diet—low in antioxidants and contaminated with mycotoxins and nitrosamines; and the oral microbiome, are still emerging. [1,2] High case fatality rates, approaching 90%, result from delayed diagnosis and major trauma associated with esophageal surgery. Unlike in many other cancers, hereditary forms are rare, there is no clear premalignant stage, and early stages of the disease are asymptomatic. Moreover, the first clinical symptoms are nonspecific and easy to overlook. Consequently, the ESCC is mostly diagnosed at an advanced stage, precluding curative tumor resection. Surgery remains the only curative modality but constitutes a challenge owing to the fact that esophagus spans three anatomic compartments and is located in close proximity to several vital organs [2]. Better understanding of molecular landscape facilitating development of targeted treatment strategies and biomarker discovery is prerequisite for improving prognosis of ESCC patients. Still, the approach, referred to as “precision/personalized medicine“, is only starting to be implemented in ESCC, and first potential therapy targets have recently been identified using next-generation sequencing (NGS) [3].

Metabolic reprogramming is one of eight recognized cancer hallmarks [4], allowing increasing demands of intensively proliferating cancer cells for energy and intermediates to be met. There is growing awareness that the process is highly dynamic. Changes in the metabolic make-up of cancer cells in localized primary tumors are distinct from those necessary to facilitate cancer cell dissemination and growth in distant organs [5]. Our preliminary research has shown that ESCC might be locally characterized by upregulated expression of genes encoding glucose transporter 1 (GLUT1) as well as inducible nitric oxide synthase (NOS) and ornithine decarboxylase (ODC) of the L-arginine/nitric oxide (NO)/polyamine pathway [6]. In the present study, we conducted a comprehensive analysis of the L-arginine/NO pathway status at the local transcriptomic and systemic metabolic level, in order to screen for potential molecular therapy targets and biomarkers, respectively. Alterations in the pathway metabolites as well as their suitability as diagnostic tools have recently been demonstrated by our group in colorectal cancer (CRC) [7] and inflammatory bowel disease, the condition associated with increased CRC risk [8].

L-arginine, further referred to as arginine, is a conditionally essential amino acid and a source of various biologically active metabolites, including NO—synthesized by NOS enzymes and ornithine—synthesized by arginases (ARG) and used by ODC for the production of polyamines. Arginine plays a dual role in cancer. On the one hand, it is necessary for immune cells to fight against the disease. Its accelerated uptake and metabolism by cancer cells is a strategy of immune evasion and, therefore, arginine supplementation is viewed as an antineoplastic therapeutic approach. On the other hand, both arginine and its metabolic products, particularly polyamines, facilitate tumor growth and metastasis [9,10,11,12]. It has been demonstrated in a mice model of breast cancer that a shift towards arginase and polyamine synthesis is a hallmark of early metastatic disease [13]. Exploiting cancer requirement for arginine by upregulating endogenous or supplementing exogenous arginine-consuming enzymes is considered for arginine-auxotrophic cancers [11,12].

**Figure 1:**
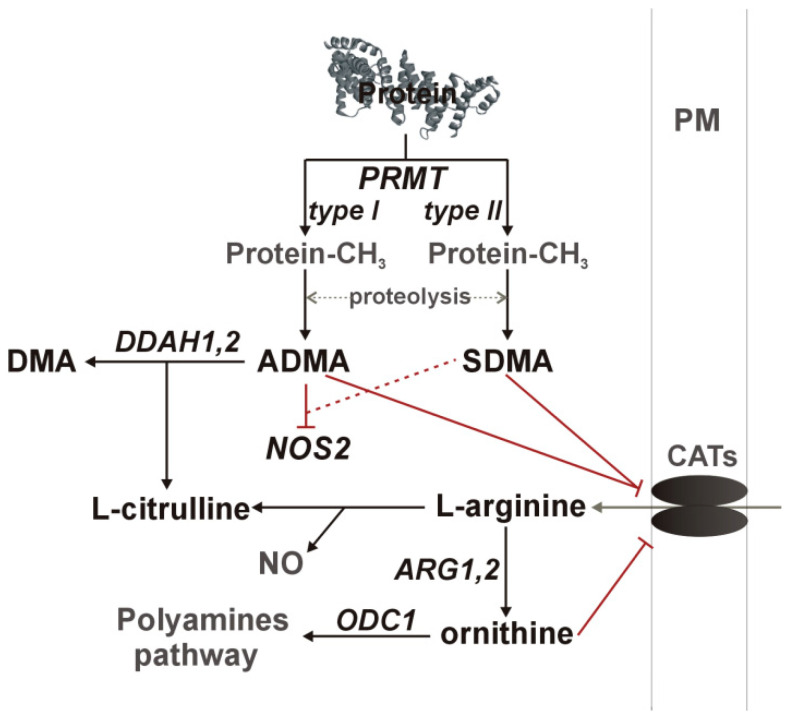
A simplified overview of the L-arginine/NO pathway. Pathway enzymes are written in italics and metabolites in a straight script; gray color was used to indicate pathway components not determined in the current study. Inhibitory effects are marked in red, with dashed line if the effect is weak. ADMA, asymmetric dimethylarginine; ARG, arginase; CATs, cationic amino acid transporter; DDAH, dimethylarginine dimethylaminohydrolase; DMA, dimethylamine; NOS, nitric oxide synthase; ODC, ornithine decarboxylase; PM, plasma membrane; PRMT, protein methyltransferase.

Nitric oxide synthesis by NOS enzymes is regulated by arginine availability, controlled by asymmetric (ADMA) and symmetric dimethylarginine (SDMA) and ornithine at the level of cationic amino acid transporter (CAT-1), as well as by enzyme inhibition by ADMA and, to a much lesser degree, by SDMA. Dimethylarginines are products of degradation of methylated proteins. Methyl groups are attached by arginine N-methyltransferases (PRMT), consisting of type I enzymes (e.g., PRMT1), which yield ADMA after proteolysis and type II enzymes (e.g., PRMT5), which yield SDMA. SDMA is mainly excreted with urine while ADMA is catabolized by dimethylarginine dimethylaminohydrolase (DDAH) into citrulline and dimethylamine (DMA) [9,10,11,12,14]. A schematic representation of the L-arginine/NO pathway is depicted in Figure 1.

In view of a pressing need for new therapeutic targets and biomarkers, the aim of the present study was to investigate the L-arginine/NO pathway status in ESCC. We analyzed the pathway metabolites (arginine, citrulline, ornithine, ADMA, SDMA, and DMA) at the systemic level, referring them to ESCC advancement and putting them into the broad context of the inflammatory, immune and angiogenic milieu. Two indices, surrogate indicators of arginine general bioavailability (arginine-to-(citrulline+ornithine) ratio) and of its availability for NO synthesis (arginine-to-ADMA ratio), were calculated as well. We also determined the suitability of pathway metabolites and metabolite-derived indices as differential markers in ESCC. In addition, the local expression of key pathway enzymes (*ARG1*, *ARG2*, *DDAH1*, *DDAH2*, *NOS2*, *ODC1*, *PRMT1*, and *PRMT5*), as potential therapeutic targets, was evaluated in reference to cancer pathology.

## 2. Results

### 2.1. Systemic Concentrations of L-arginine/NO Pathway Metabolites

#### 2.1.1. L-arginine/NO Pathway Metabolites in ESCC and Benign Conditions

**Figure 2:**
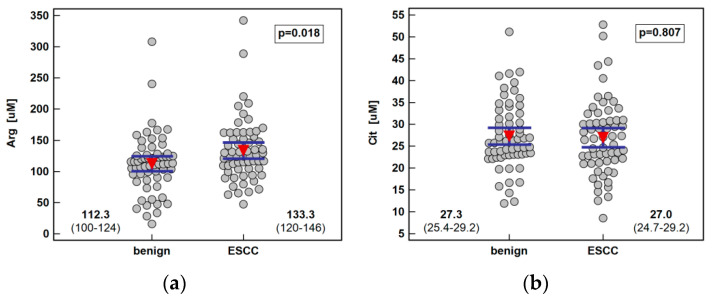

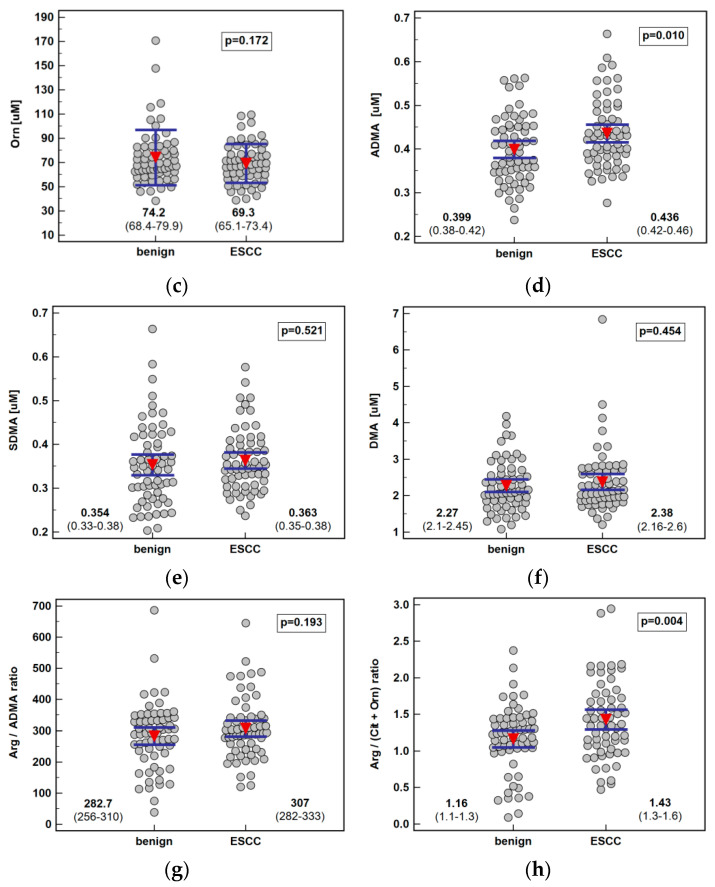
Systemic concentration of L-arginine/NO pathway metabolites in patients with esophageal squamous cell carcinoma (ESCC; *n* = 61) and individuals with benign conditions of the esophagus (*n* = 62): (**a**) arginine (Arg); (**b**) citrulline (Cit); (**c**) ornithine (Orn); (**d**) asymmetric dimethylarginine (ADMA); (**e**) symmetric dimethylarginine (SDMA); (**f**) dimethylamine (DMA); (**g**) arginine-to-ADMA ratio (Arg/ADMA); (**h**) arginine-to-(citrulline+ornithine) (Arg/(Cit+Orn). Data presented as the means with 95% confidence interval (red triangles with whiskers as well as numeric data) and analyzed using a *t*-test for independent samples.

Systemic concentration of the pathway metabolites was determined using LC-QTOF-MS (liquid chromatography quadrupole time-of-flight mass spectrometry) in 61 patients with ESCC and 62 individuals with benign conditions of the esophagus. Both groups were well age and sex matched. The ESCC patients had higher arginine and ADMA than individuals with benign conditions of the esophagus. There was no significant difference in the Arg/ADMA ratio, indicative of arginine availability for NO synthesis, but the general arginine bioavailability was greater in ESCC as indicated by the higher Arg/(Cit+Orn) ratio. The other metabolites, that is, citrulline, ornithine, SDMA, and DMA, did not differ between groups (Figure 2).

There were 16 patients with resected tumors of esophagus admitted three months postsurgery for the esophagoplasty. We compared the systemic concentration of metabolites between those patients and the ESCC group and found that ESCC patients tended to have higher arginine (124.8 µM (116–135) vs. 112.4 µM (88–125), *p* = 0.058) and ADMA (0.43 µM (0.40–0.44) vs. 0.38 µM (0.31–0.45), *p* = 0.081) than esophagoplasty patients. In turn, esophagoplasty patients were distinguished by a significantly higher concentration of SDMA than ESCC patients (0.42 µM (0.35–0.44) vs. 0.35 µM (0.33–0.38), *p* = 0.025).

#### 2.1.2. Association of L-arginine/NO Pathway Metabolites with ESCC Advancement

**Figure 3:**
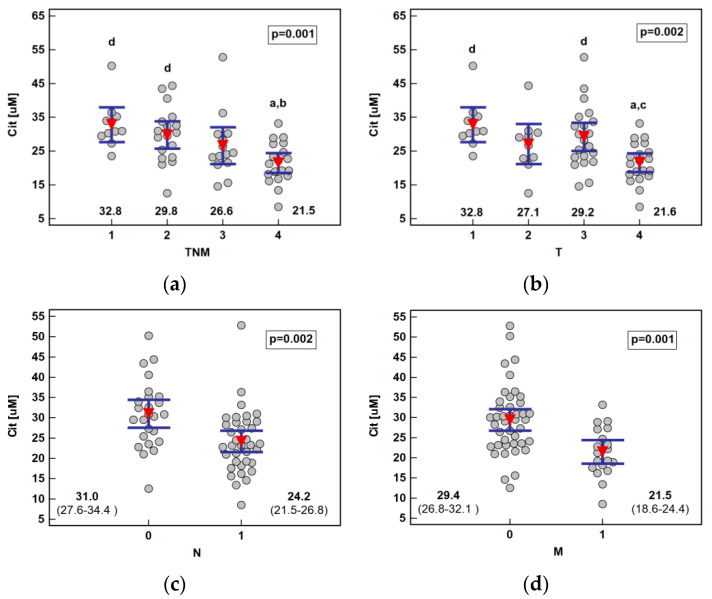
Association between systemic citrulline (Cit) concentration and ESCC advancement: (**a**) tumor-node-metastasis (TNM) stage; (**b**) primary tumor extension (T); (**c**) lymph node involvement (N); (**d**) distant metastasis (M). Data presented as the means with 95% confidence interval (red triangles with whiskers as well as numeric data) and analyzed using one-way ANOVA. a, significantly different from T1; b, significantly different from T2; c, significantly different from T3; d, significantly different from T4.

The analysis of the metabolite concentration against ESCC advancement showed that only citrulline was significantly associated with the disease overall stage (TNM) as well as all its components: primary tumor extension, lymph node involvement, and distant metastasis. The metabolite concentration dropped along with increasing stage (ρ = −0.55, *p* < 0.0001), being significantly lower in stage IV than I or II, and along with primary tumor extension (ρ = −0.47, *p* = 0.0001), being significantly lower in T4 than T1 or T3. Citrulline was significantly lower in ESCC patients with lymph node or distant metastases (Figure 3).

**Figure 4:**
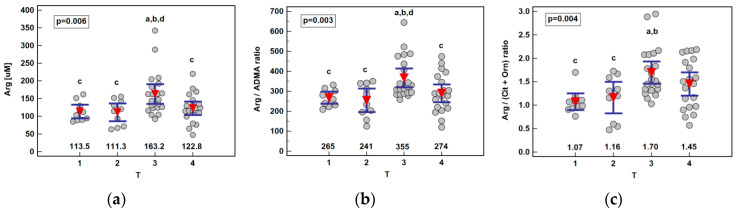
Association between systemic arginine concentration or arginine-based ratios and primary tumor extension (T) in patients with esophageal squamous cell carcinoma: (**a**) arginine (Arg); (**b**) arginine-to-ADMA ratio (Arg/ADMA); (**c**) arginine-to-(citrulline+ornithine) (Arg/(Cit+Orn)). Data presented as the means with 95% confidence interval (red triangles with whiskers as well as numeric data) and analyzed using one-way ANOVA. a, significantly different from T1; b, significantly different from T2; c, significantly different from T3; d, significantly different from T4.

Except for arginine elevation in patients with T3 cancers, no other metabolite was significantly associated with the disease advancement. Consequently, arginine-based ratios, Arg/ADMA and Arg/(Cit+Orn), were significantly higher in T3 cancers as well (Figure 4). Owing to the gradual citrulline depletion with the disease advancing, Arg/(Cit+Orn) correlated with TNM (ρ = 0.27, *p* = 0.036) and T (ρ = 0.29, *p* = 0.023). The association between the arginine and T stage remained significant (*p* = 0.029) following the removal of two outlying observations in T3 cancers, although the difference between T2 and T3 lost significance.

#### 2.1.3. L-arginine/NO Pathway Metabolites as Differential Markers

**Figure 5:**
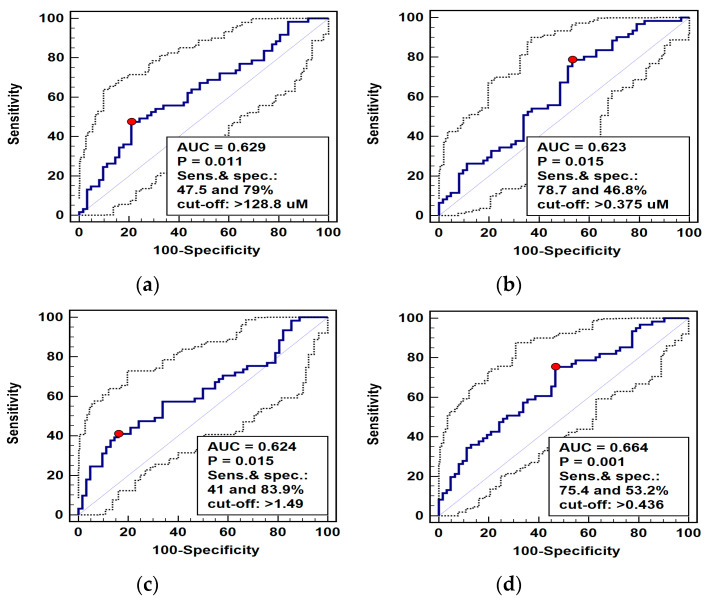
Receiver operating curve (ROC) analysis of diagnostic potential of L-arginine pathway metabolites in differentiating esophageal squamous cell carcinoma (ESCC) from benign conditions: (**a**) arginine (Arg); (**b**) asymmetric dimethylarginine (ADMA); (**c**) arginine-to-(citrulline+ornithine) ratio (Arg/(Cit+Orn)); (**d**) panel consisting of ADMA and Arg/(Cit+Orn). Graphs present ROC curve (solid curve) with 95% confidence interval (dotted curves) and an optimal cut-off value (red dot). Performance of a chance marker devoid of diagnostic power (AUC = 0.5) is indicated by a diagonal line. AUC, area under ROC curve; Sens., sensitivity; spec., specificity.

Pathway metabolites, which were significantly different in patients with ESCC and benign esophageal conditions, were analyzed as potential differential markers using receiver operating curve (ROC) analysis. The individual performance of the arginine, ADMA, and Arg/(Cit+Orn) ratio was very comparable, although arginine and Arg/(Cit+Orn) had superior specificity and ADMA—sensitivity. Logistic regression (stepwise method) was applied to identify the independent predictors of ESCC. The ADMA and Arg/(Cit-Orn) were selected, and predicted probabilities were used in a ROC curve analysis to assess the diagnostic power of their concomitant assessment. The overall accuracy of the ADMA and Arg/(Cit-Orn) panel improved only minimally (Figure 5).

#### 2.1.4. Interrelationship between Systemic Concentrations of L-arginine/NO Pathway Metabolites

The interrelationship between systemic concentrations of L-arginine/NO pathway metabolites in ESCC patients and individuals with benign conditions of the esophagus was compared (Table 1). ADMA was positively correlated with all other metabolites in both ESCC and patients with benign conditions; although in ESCC, the correlation was stronger with arginine and SDMA and in patients with benign conditions with citrulline. Arginine was correlated positively with citrulline in both groups but with SDMA only in ESCC. Citrulline correlated with arginine and tended to with ADMA in ESCC; while in benign conditions, its correlation with ADMA was stronger, and there was positive correlation with DMA as well. DMA was the most strongly correlated with SDMA in both groups and with ADMA in ESCC rather than with citrulline like in the benign group. Ornithine correlated weakly with ADMA in both groups and with SDMA in ESCC patients. SDMA was correlated with ADMA and SDMA in both groups but only with arginine and ornithine in ESCC.

#### 2.1.5. Interplay Between L-arginine/NO Pathway Metabolites and Circulating Cytokines and Growth Factors

Arginine was rather poorly interrelated with circulating inflammatory, immune, and angiogenic mediators. There was a fair positive correlation with hepatocyte growth factor (HGF) and tumor necrosis factor (TNF)-related apoptosis inducing ligand (TRAIL) and a slightly weaker association with TNFα, monocyte chemoattractant protein (MCP)-1, platelet-derived growth factor (PDGF)-BB, and stem cell factor (SCF). Ornithine displayed a fair negative correlation with IL-7 and a positive one with macrophage migration inhibitory factor (MIF). In turn, citrulline was tightly, although negatively, correlated with a number of circulating cytokines and growth factors. It displayed a moderate inverse correlation with IL-13 and fair negative correlations with the following cytokines (ordered by decreasing strength): interferon (IFN)-γ, IL-10 and macrophage inflammatory protein (MIP)-1β, granulocyte-macrophage colony-stimulating factor (GM-CSF) and IL-5, IL-17 and TNFα, fibroblast growth factor (FGF)2, granulocyte colony-stimulating factor (G-CSF) and IL-1β, IL-1ra and IL-8, IL-4, MIF, IL-6, IL-12p70 and MIP-1α, IL-15, eotaxin, and leukemia inhibitory factor (LIF) (Table 2).

The ADMA displayed the strongest fair positive correlation with growth-regulated alpha protein (GROα), followed by TRAIL, IFNα2, IL-3 and interleukin 2 receptor subunit α (IL-2Rα), stromal cell-derived factor (SDF)-1α, C-C motif chemokine ligand 27 (CTAK), and nerve growth factor β (β-NGF) and HGF. The SDMA was more tightly related to circulating cytokines. It was the most strongly correlated with SCF and displayed a fair positive correlation also with CTAK, LIF, IL-3, IFNγ-induced protein 10 (IP-10), MIP-1α, and stem cell growth factor β (SCGF-β), β-NGF and GROα, IFNα2, SDF-1α, IL-13, HGF and monokine induced by gamma interferon (MIG), GM-CSF and IL-15, IL-16, and MIP-1β. The DMA displayed a moderate positive correlation with SCF and a fair correlation with SCGF-β, TRAIL, IL-16, HGF, IL-3, IL-2Rα and LIF, GROα and IFNα2, β-NGF and IL-19, and MIP-1β and SDF-1α (Table 2).

The arginine bioavailability index (Arg/(Cit+Orn)) correlated positively with IL-17, followed by IL-7, CTAK, IL-1β, and TNFα and negatively with MIF. Arginine availability for NO synthesis (Arg/ADMA) was positively related to PDGF-BB concentration and negatively to vascular endothelial growth factor (VEGF)-A and MIF (Table 2).

### 2.2. Transcriptional Analysis of Local Expression of Key L-arginine/NO Pathway Enzymes

#### 2.2.1. Pairwise Analysis of Enzyme Expression in Tumor and Adjacent Tissue

Quantitative (real-time) polymerase chain reaction (PCR) with SYBR Green chemistry was used to determine the relative expression level of *ARG1* and *ARG2*, *DDAH1* and *DDAH2*, *NOS2*, *ODC1*, and *PRMT1* and *PRMT5* in 40 patient-matched samples from esophageal tumors and adjacent, macroscopically normal mucosa. 

**Figure 6:**
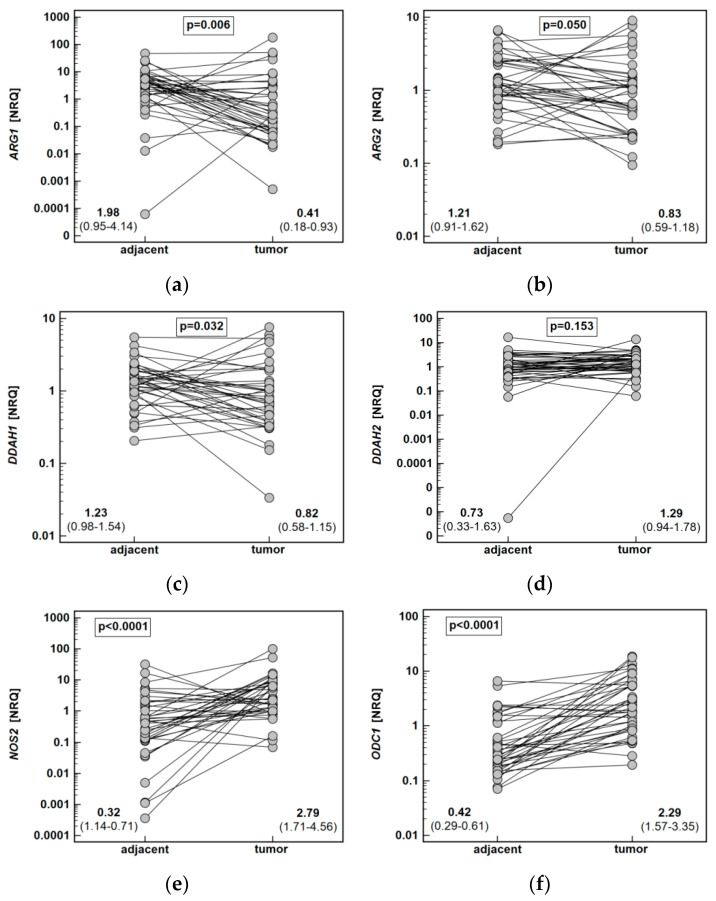

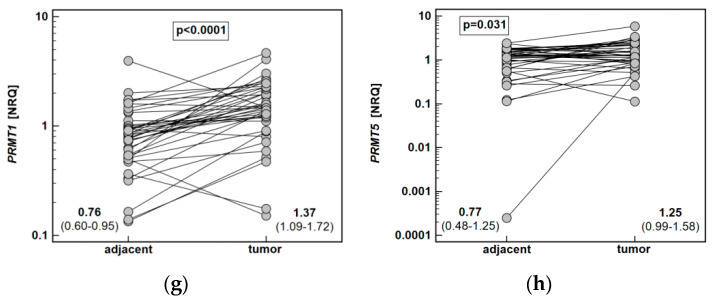
Tumor and noncancerous tumor-adjacent tissue expression of key enzymes of the L-arginine/NO pathway: (**a**) *ARG1*; (**b**) *ARG2*; (**c**) *DDAH1*; (**d**) *DDAH2*; (**e**) *NOS2*; (**f**) *ODC1*; (**g**) *PRMT1*; (**h**) *PRMT5*. Data analyzed using a *t*-test for paired samples and presented as means of normalized relative quantities (NRQ) with 95% confidence interval. ARG, arginase; DDAH, dimethylarginine dimethylaminohydrolase; NOS, nitric oxide synthase; ODC, ornithine decarboxylase; PRMT, protein arginine N-methyltransferase.

Both *ARG1* (by 4.9-fold) and *ARG2* (1.5-fold) were significantly downregulated in tumors as compared to adjacent tissue. The *DDAH1* was downregulated as well (by 1.5-fold), while *DDAH2* expression did not differ. The *NOS2* (by 8.7-fold) and *ODC1* (5.4-fold) expression was upregulated in tumors. Likewise, *PRMT1* (by 1.8-fold) and *PRMT5* (by 1.6-fold) expression in tumors was higher than in noncancerous adjacent mucosa (Figure 6). 

#### 2.2.2. Association between Fold Change in Enzyme Expression and ESCC Pathology

Potential relationship between fold change in enzyme expression (tumor-to-adjacent) and ESCC pathology was examined (Table 3). 

Only the fold change in *DDAH2, ODC1,* and *PRMT5* expression differed significantly with overall TNM stage, with tumor-to-adjacent ratio decreasing in a stepwise manner along with increasing stage. The same pattern was present for an association between depth of tumor invasion (T) and fold change in *DDAH2* and *ODC1*. Fold change in *ARG2* (by 1.9-fold), *DDAH2* (by 3.8-fold), *NOS2* (by 5.0-fold), *ODC1* (by 2.2-fold), and *PRMT5* (by 2.3-fold) tended to decrease, and that of *PRMT1* was significantly lower (by 1.6-fold) in cancers metastasizing into lymph nodes (N≥1). Fold change in *ODC1* was significantly lower (by 4.1-fold) in cancers metastasizing to distant organs as well (Table 3). 

We investigated whether inverse relationship between expression ratios (tumor-to-adjacent) and cancer advancement results from changes in gene expression in tumor (Table 4) or/and in adjacent noncancerous tissue (Table 5).

This analysis revealed that a negative correlation between *ODC1* expression ratio and TNM resulted from the concomitant downregulation of enzyme expression in tumors along with the increasing stage (Table 4) and its upregulation in noncancerous adjacent tissue (Table 5). The expression of *DDAH2* did not change in tumors but tended to increase along with increasing TNM in the adjacent tissue (Table 5), contributing to a significantly negative correlation between expression rate and stage (Table 3). Additionally, *ARG2* expression in adjacent tissue tended to increase with advancing disease (Table 5).

The clear tendency of expression ratios being lower in N≥1 than N0 cancers also seem to result from combined slightly lower enzyme expression in tumors (Table 4) and higher in adjacent noncancerous tissue (Table 5) derived from of N≥1 patients.

Only lower *ODC1* expression ratio in M1 than M0 cancers (Table 3) resulted from significantly lower gene expression in tumors from M1 patients (by 3.5-fold) (Table 4). The presence of distant metastases was, in turn, associated with significantly higher expression of *NOS2* in tumors (by 5.1-fold) (Table 4). It was also associated with a tendency towards a higher expression of *ARG1* and *DDAH1* in noncancerous tumor-adjacent tissue (Table 5).

#### 2.2.3. Interrelationship between Local Expression Levels of L-arginine/NO Pathway Enzymes

Correlation patterns in the enzyme expression in tumor (Table 6) and noncancerous adjacent tissue were compared (Table 7). In tumor tissue, both *ARG*s were correlated positively with both *DDAH*s, while in adjacent tissue, *ARG1* was correlated solely with *DDAH1*. In tumors, *NOS2* correlated with both *PRMT*s, while in adjacent tissue, only with *PRMT1* and additionally with *ODC1*. In tumors, *ODC1* correlated with both *ARG*s, *DDAH2*, and *PRMT5,* while in adjacent tissue, solely with *ARG2* isoenzyme, with *DDAH2*, *NOS2*, and both *PRMT*s. The expression pattern for *DDAH1* was similar, although the associations with *ARG1* and *PRMT*s were weaker in tumors. In turn, *DDAH2* in tumors was positively correlated with all genes, while in adjacent tissue, there was no correlation with *ARG1* and *NOS2*. The expression of *PRMT5* in tumors correlated with *NOS2* but not *ARG1*, while in adjacent tissue, with *ARG1* but not *NOS2*. The expression of *PRMT1* in tumors did not correlate with *ODC1*, while the association was present in tumor-adjacent tissue. 

#### 2.2.4. Correlation between Local Expression Levels of L-arginine/NO Pathway Enzymes and Circulating Cytokines, Chemokines, and Growth Factors as Well as Systemic Concentrations of Pathway Metabolites

The correlation pattern between enzyme expression in tumor (Table 8) and noncancerous tumor-adjacent tissue (Table 9) and systemic concentrations of cytokines, chemokines, and growth factors was examined (*n* = 36). There was a negative correlation between *PRMT1* expression and ADMA in tumors. Also, *ARG1* tended to positively correlate with ADMA and arginine and *ARG2* with citrulline and SDMA. Ornithine negatively correlated with *DDAH1* (Table 8). In noncancerous tissue, ADMA tended to correlate with *ARG1* (Table 9).

Regarding circulating cytokines, *ARG1* in tumors positively correlated with HGF and IL-18 and *ARG2* with SDF-1α. *DDAH1* displayed a negative correlation with FGF2, while *DDAH2* did not show any significant associations. *NOS2* positively correlated with IL-4, IL-5, and IL-6 and *ODC1* with MCP-1. No significant associations could be observed for *PRMT*s (Table 8).

In noncancerous tissue, *ARG1* was inversely related to HGF and IL-2Rα and *ARG2* positively with MIP-1β. *DDAH2* positively correlated with IL-9, IP-10, and SCGFβ, *NOS2* with IL-3, IL-9, and LIF, and *ODC1* with IL-18, LIF, MIP-1β, RANTES, and SCF. Both *PRMT*s positively correlated with MIP-1β. In addition, *PRMT5*correlated with IL-5 and IL-7 (Table 9).

## 3. Discussion

In the era of precision medicine, metabolic reprogramming of cancer cells is considered a promising source of potential biomarkers and novel molecular targets for antineoplastic strategies [15]. In this respect, the L-arginine/NO pathway becomes an area of active investigation. Its focus is shared between the arginine/ornithine/polyamines rout, the interest in which has recently rekindled, and arginine methylation, its methylated derivatives, and their metabolism, an understudied topic in cancer context. The present study provides a comprehensive overview of pathway status in ESCC, including local enzyme expression and systemic metabolites, with reference to a broad spectrum of inflammatory, immune, and angiogenic mediators. Corroborating our own preliminary observations [6] as well as findings of others [16,17], *ODC1* expression in ESCC tumors was markedly upregulated as compared to adjacent noncancerous tissue. As a rate-limiting enzyme of the polyamine pathway and a down-stream target of c-Myc, ODC is implicated in facilitating tumor growth [18]. Moreover, its overexpression can induce neoplastic transformation, making *ODC1* an oncogene [19]. Accordingly, *ODC1* expression in the colon is upregulated in conditions associated with increased risk of cancer [8]. In turn, the enzyme inhibitors used as chemoprevention, alone or in combination with nonsteroidal anti-inflammatory drugs, have shown promising results in animal models of CRC [20] as well as in clinical trials [18,21]. They reduced the number and size of tumors in test animals or prevented formation of sporadic adenomas, respectively. The enzyme has recently been shown to promote ESCC as well. It induces cell proliferation and survival, as the gene silencing or enzyme inhibition with the substrate analogue, difluoromethylornithine, results in cell arrest in the G2/M phase and triggers apoptosis [17]. The advocated enzyme involvement in the initial phases of carcinogenesis would explain a significant enzyme upregulation in tumors and yet a negative correlation of expression rate with ESCC advancement observed here. However, He et al. [17] reported ODC protein to be more pronouncedly expressed in samples obtained from patients with stage III or N1 cancers than with stage II or N0. When *ODC1* expression patterns were inspected more thoroughly, we found that *ODC1* expression in patients with more advanced cancers decreased in tumors but increased in adjacent tissue. As the changes affect both normal tissue and the tumor, and they occur in opposite directions, unsurprisingly, systemic ornithine did not differ with respect to ESCC stage.

Likewise, a negative correlation between the disease advancement, particularly lymph node involvement, was observed for *DDAH*s and *PRMT*s. Similarly to *ODC1*, it resulted from gene downregulation in tumors with concomitant upregulation in tumor-adjacent noncancerous tissue. The phenomenon of so-called “tumor molecular margin”, describing alterations in molecular landscape in the tissue surrounding tumor, is increasingly recognized and has previously been documented also in ESCC [22]. This phenomenon is of clinical relevance as it is held responsible, at least in part, for synchronous tumors and cancer recurrence following treatment [23,24]. Moreover, it is considered to better represent dysregulations leading to neoplastic transformation than changes encountered in already transformed tissue. As such, studying the tumor molecular margin is deemed better suited to aid the discovery of potential targets for chemoprevention [24]. Regarding L-arginine/NO pathway enzymes, our group showed *DDAH*s and *PRMT*s in CRC to be apparently downregulated, as their relative expression was lower in tumors than in adjacent tissue, while they were in fact upregulated in both. There were no normal esophageal mucosa samples available for comparison in the present study, but the observation on adjacent tissue not being “inert” but displaying an increasing enzyme expression with advancing disease seems to indicate that ESCC might be like CRC in this respect. Still, a lack of control mucosa samples from healthy individuals should be recognized as a limitation of the current study.

The analysis of RNA-seq expression data gathered by The Cancer Genome Atlas (TCGA) Research Network and the Genotype-Tissue Expression (GTEx) project indicated *DDAH1* upregulation in ESCC [25], although confirming individual studies, analyzing *DDAH1* association with reference to ESCC pathology, seem to be missing. The *DDAH2* status, as well as the precise role of either isoform in cancer, remains largely unknown. Here, *DDAH1* was slightly downregulated in tumors, and *DDAH2* did not show significant difference as compared to the surrounding tissue. Their expression levels were interrelated, but there was variation in their correlation pattern with other genes as well as circulating cytokines, supporting the notion that neither their distribution nor function is completely overlapping [25]. Both enzymes catabolize ADMA, but their spatial transcriptional patterns differ and they may be altered in cancer in a different, sometimes opposing, manner [25]. The overexpression of DDAH1 and DDAH2 is linked with the promotion of angiogenesis by the indirect stimulation of VEGF-A expression and NO synthesis, via degrading NOS inhibitor—ADMA. Consequently, their targeting is viewed as an emerging anticancer strategy [25,26]. However, neither *DDAH1* nor *DDAH2* correlated with circulating VEGF-A in the present study. On the contrary, *DDAH1* in tumors exhibited a negative correlation with another angiogenic factor, FGF2, and *DDAH2* in tumor-adjacent tissue—a positive association with an angiostatic IP-10. Moreover, the expression of *DDAH2* only tended to weakly correlate with *NOS2* in tumors, although in prostatic cancer cell lines, its expression was accompanied by the upregulation of iNOS and VEGF-A [27]. As mentioned, ADMA is considered to inhibit angiogenesis, but its role in cancer is unclear. Extensively investigated in cardiometabolic diseases [28], it has been understudied in cancer. However, limited evidence indicates that ADMA accumulate in tumor and surrounding tissue [29] and may support tumor growth by protecting cancer cells from nutritional stress and drug-induced death [30]. Here, consistently with a cancer-promoting role, its systemic concentrations were elevated. Moreover, ADMA levels positively correlated with immune mediators known to support cancer development, the most strongly with GROα (CXCL1), the cytokine released from tumors as well as tumor-associated macrophages and implicated in the recruitment of tumor-associated neutrophils [31] and promotion of metastasis [32], respectively, and associated with poor overall survival [33].

The expression of various PRMT isoenzymes in cancer is also largely unknown, but gaining attention as potential targets for chemoprevention [34,35]. Their targeting is also investigated as potential strategy for overcoming cancer chemoresistance [36]. It becomes increasingly apparent that the role of PRMTs is not limited to NO synthesis. The enzymes have been implicated in the global regulation of RNA splicing and translation [37]. Both PRMT1 and PRMT5 support tumor growth and are, therefore, overexpressed in a number of cancers [34]. Corroborating our observation, the expression of PRMT1 is upregulated in ESCC [38,39] and head and neck tumors [40]. Mechanistically, it facilitates cell proliferation and migration of oral and esophageal squamous cell carcinoma cells [38,40]. In addition, Zhao et al. [39] showed PRMT1 to be preferentially expressed in esophageal tumor initiating cells and function to enhance the self-renewal features, tumorigenicity, and chemoresistance of ESCC. The role of PRMT5 in cancer is even more poorly understood. Mostly oncogenic, owing to promoting cancer cell proliferation and migration, in breast cancer it prevents metastasis, while data regarding prostate cancer are contradictory (reviewed in [41]). Only recently, PRMT5 has been shown to regulate Hsp90A, a known cancer-related chaperone providing protection for a number of oncoproteins [42]. There seems to be no previous reports on PRMT5 expression in ESCC. We found it upregulated in tumors as compared to adjacent tissue. In cancers metastasizing to lymph nodes, its expression increased in tumor adjacent tissue. The expression of *PRMT5* was tightly associated with that of *PRMT1*, more so in adjacent tissue than tumors. Of the other pathway enzymes, *PRMT5* expression was associated closely with that of *ARG2* and *DDAH*s, in tumors more so with *DDAH2* than *DDAH1*. In turn, a correlation with *NOS2* could be observed in ESCC tumors. Reflecting enzyme ability to induce expression of proinflammatory genes [43], both *PRMT*s were positively correlated with circulating MIP-1β.

Arginases compete with NOS enzymes for arginine and, as NOS activity is considered cancer-promoting, strategies based on upregulating endogenous arginase activity or introduction of exogenous arginine-consuming enzymes are currently being tested [11,12]. Moreover, arginases synthesize ornithine and, therefore, supply substrates for polyamine biosynthesis [44]. Accordingly, *ARG2* expression level, and in tumors also that of *ARG1*, was positively correlated with *ODC1*, a key regulatory enzyme of the pathway. However, arginine is necessary for the proper functioning of immune cells, and its depletion is a strategy of immune evasion employed by cancer cells [11,45,46]. Moreover, arginase-2 is expressed by immunosuppressive M2 polarized macrophages and dendritic cells as well as myeloid-derived suppressor cells [47]. In the sample set investigated, *ARG1* was substantially while *ARG2* borderline significantly downregulated in tumors. Consistent with counter-regulatory mechanisms in the ARG/NOS interplay [44], *NOS2* was markedly upregulated and *ARG1* tended to positively correlate with the systemic concentration of ADMA, a NOS inhibitor. Moreover, *NOS2* was the only pathway enzyme clearly expressed at a higher level in metastasizing tumors. In line with its proinflammatory character, *NOS2* expression correlated positively with circulating immune and inflammatory mediators, whilst *ARG1*, a hepatocyte isoform [44], correlated with HGF, positively in tumors but negatively in adjacent tissue. Consistent with this observation, HGF/c-met signaling axis was shown to induce macrophage M2 polarization as its inhibition upregulated *NOS2* and other M1 polarization markers and downregulated *ARG1* [48]. The expression of *ARG2*, but not *ARG1*, has been shown to be upregulated by hypoxia and implicated in mediating hypoxia-induced cancer cell proliferation [49]. Moreover, cancer-associated fibroblasts and tumor-associated M2 macrophages from hypoxic regions overexpress ARG2 as well [50]. In macrophages, HIF-1α controls iNOS while HIF2α—arginase [51]. Supporting the ARG2 link with hypoxia and angiogenesis, *ARG2* expression in evaluated tumors positively correlated with SDF-1α, a hypoxia-induced proangiogenic factor [52], although its relation to VEGF-A tended to be negative.

Cancer-associated upregulation of arginase -1 contributes to a diminished extracellular arginine pool [11]. Here, consistently with decreased *ARG1* and *ARG2* expression, systemic arginine was elevated. Likewise, the arginine-to-citrulline and ornithine ratio was higher, indicative of increased arginine bioavailability in ESCC. A weak positive correlation between amino acid bioavailability and the disease stage resulted from gradually decreasing citrulline concentration, implying accelerating activity of the arginine-citrulline pathway in parallel with ESCC advancement. Arginine can be regenerated from citrulline by argininosuccinate synthetase (ASL) and argininosuccinate lyase (ASS1), and ASS1 is a rate-limiting enzyme of this pathway [53]. As *ASS1* expression, especially leukocyte enzyme, is controlled by inflammatory and immune mediators [53], the systemic citrulline concentration in our patients was tightly but inversely related to their level. The expression of *ASS1* is upregulated also by p53, in order to ensure cell survival under genotoxic stress conditions [54]. Still, *ASS1* is reported to be epigenetically silenced in some cancer types [55], making them sensitive to antineoplastic therapy based on arginine-deprivation. Nonaffected arginine bioavailability, due to the efficient arginine-citrulline recycling pathway demonstrated here, would indicate that ESCC is not a suitable candidate.

Elevated plasma concentrations of arginine, dimethylarginines (pooled ADMA and SDMA), and N-acetylated derivatives of putrescine and ornithine have been observed in children with eosinophilic esophagitis [56]. In line with the implicated close relationship between eosinophils and the pathway metabolites, both *ARG*s tended to positively correlate with IL-3 in tumors. The expression of *ARG1* in adjacent tissue also correlated with other eosinophil-associated cytokines such as IL-13 and GM-CSF and that of *NOS2* with IL-5. In addition, ADMA positively correlated with IL-3 and SDMA also with IL-13 and GM-CSF.

Correlation analysis showed that *DDAH2*, *NOS2*, and *ODC1* expression in normal tissue was positively correlated with circulating LIF. In addition, LIF concentration was correlated also with SDMA and, consistently with a possible positive effect on *DDAH2*, with DMA. The cytokine is regarded as an oncogene, facilitating the self-renewal of tumor-initiating cells, supporting cancer-associated fibroblasts, and inducing resistance to radio- and chemotherapy [57,58,59]. It has also been found to hamper anti-PD1 therapy by upregulating MIG in tumor-associated macrophages [60]. Consistently, the ADMA and SDMA concentration in our ESCC patients was correlated with that of MIG as well. Intriguingly, various components of the L-arginine/NO pathway were related to the factors associated with cancer cell renewal. The expression of *ODC1* was associated with SCF, as was arginine and, more so, SDMA and DMA concentration. The SCF and its receptor c-Kit (CD117) ensure cancer stem cell viability and self-renewing properties. It might be membrane-bound and expressed in cancer cells or tumor-associated macrophages and cancer-associated fibroblasts, from which a soluble form of SCF can be released [61]. The expression of *DDAH2* and systemic SDMA and DMA were, in turn, associated with circulating SCGFβ, a growth factor for primitive hematopoietic progenitor cells, which is overexpressed in cancer by circulating cancer cells [62] and drug-resistant cancer stem cells [63].

Metabolomics is gaining interest as a potential tool in biomarker discovery [64], especially in that it enables the concomitant quantification of a predetermined panel of metabolites. The utility of individual intermediates of the L-arginine/NO pathway as biomarkers for various conditions has repeatedly been demonstrated [56,65,66,67]. In cancer, the simultaneous quantification of SDMA, citrulline, and DMA proved excellent in detecting CRC, and the changes in the early postoperative period in arginine, ADMA, and SDMA were an accurate marker of surgical complications [7]. In ESCC, however, neither individual metabolites nor a panel consisting of ADMA and Arg/(Cit+Orn) index possessed satisfactory diagnostic power to be of clinical use.

## 4. Materials and Methods

### 4.1. Sample Collection

Biobanked material, collected in the Department of Gastrointestinal and General Surgery of Wroclaw Medical University between 2010 and 2015 (prior the introduction of radiotherapy as a treatment preceding surgery), was used in the present study.

Whole blood was drawn from patients by venipuncture into serum separator tubes following overnight fasting and prior to any treatment. Collected blood was clotted for 30 min at room temperature and centrifuged (1500× *g* for 10 min at room temperature). Obtained sera were aliquoted and stored at −45 °C until examination.

Patient-matched tumor and macroscopically normal mucosa were rinsed with PBS and immersed in RNAlater (Ambion Inc., Austin, TX, USA). Solution-soaked tissue samples were then stored at −80 °C until RNA isolation.

### 4.2. Study Population—Metabolomic Analysis

The study population for metabolomic analysis consisted of 123 patients admitted to the Department of Gastrointestinal and General Surgery of Wroclaw Medical University for the diagnosis and treatment of histologically confirmed ESCC (*n* = 61; curative surgery or palliative treatment) or benign esophageal conditions (*n* = 62), including esophagoplasty following esophagectomy (*n* = 16) or thermal burn (*n* = 1), achalasia (*n* = 25), stenosis (*n* = 2), esophageal lipoma (*n* = 2), and Zenker’s diverticulum (*n* = 16). Cancer patients underwent a standard diagnostic procedure, including blood work, physical examination, and imaging techniques, such as ultrasonography, computed tomography, and magnetic resonance. Cancers were staged clinically using the 7th edition of the Union for International Cancer Control TNM system. Detailed population characteristics are presented in Table 10.

### 4.3. Study Population—Transcriptomic Analysis

The study population for transcriptomic analysis consisted of 40 ESCC patients undergoing curative tumor resection in the Department of Gastrointestinal and General Surgery of Wroclaw Medical University. Patients with any severe systemic illness, with gross metastatic disease, or subjected to radio- or chemotherapy were not included. Patients were subjected to standard preoperative evaluation (blood work, physical examination, and imaging techniques, such as ultrasonography, computed tomography, and magnetic resonance). Cancers were staged pathologically using the 7th edition of the Union for International Cancer Control TNM system. In all cases, the resection margins were confirmed to be tumor-free. Detailed population characteristics are depicted in Table 11.

### 4.4. Ethical Considerations

The sample collection was approved by the Medical Ethics Committee of Wroclaw Medical University (#KB 28/2011 and #KB 784/2012). The study was conducted in accordance with the Helsinki Declaration of 1975, as revised in 1983, and informed consent was obtained from all study participants.

### 4.5. Analytical Methods

#### 4.5.1. Metabolomic Analysis

##### Chemicals

Benzoyl chloride (BCl), hydrochloride salts of unlabeled dimethylamine (D0-DMA), hexadeutero-dimethylamine (D6-DMA, declared as 99 atom % 2H), L-arginine, SDMA, ADMA, L-citrulline, L-Ornithine monohydrochloride, labeled L-Ornithine hydrochloride (3,3,4,4,5,5-D6-ornithine), and sodium tetraborate were procured from Sigma-Aldrich (Poznan, Poland). Isotope labeled L-arginine:HCl (D7-arginine, 98%) and asymmetric dimethylarginine (2,3,3,4,4,5,5-D7-ADMA, 98%) were obtained from Cambridge Isotope Laboratories (Tewksbury, MA, USA). Methanol, acetonitrile, water, and formic acid were acquired from Merck Millipore (Warsaw, Poland), and leucine–enkephalin was obtained from Waters (Milford, MA, USA).

##### LC-QTOF-MS Analysis of Selected L-arginine/NO Pathway Metabolites

The stock solutions of DMA, arginine, ADMA, SDMA, citrulline, ornithine, and isotope labeled standards were prepared in water and stored at −20 °C. Standard calibration curves were prepared by diluting the stock solutions in water in the following concentration ranges: 3.0–150 µM for ornithine, 5.0–250 µM for arginine, 0.05–2.5 µM for ADMA and SDMA, 1.0–50 µM for citrulline, and 0.14–7.0 µM for DMA.

Serum samples and calibration standards were prepared using previously validated and described methods [7,8,68,69,70,71]. Analysis of L-arginine/NO pathway metabolites as benzamide derivatives allows for the chromatographic separation of highly polar substances using a reversed phase system with relatively high retention. Importantly, it also enabled the chromatographic separation of ADMA and SDMA. The extraction and derivatization procedure was conducted as follows: 100µL aliquots of the calibration standards or serum were mixed with 10 µL internal standard solution (50 µM D6-DMA, 20 µM D7-ADMA, 100 µM D7-arginine, and 70 µM D6- ornithine, respectively) and 50 µL borate buffer (0.025 M Na_2_B_4_O_7_·10H_2_O, 1.77 mM NaOH, pH = 9.2) and vortexed (1 min, 25 °C). The samples were derivatized with 10% BCl in acetonitrile (400 µL) at 25 °C for 10 min. The mixture was centrifuged at 10,000 RPM for 7 min in 4 °C. Two microliters of supernatants diluted with water in a 1:5 ratio was injected into an LC system consisting of a Waters nanoACQUITY UPLC and a nanoAcquity HSS T3 column (C18-phase, internal diameter 1 mm, length 50 mm, particle size 1.8 μm). They were eluted at a flow rate of 0.22 mL/min with a linear gradient of 0.1% formic acid in water (A) and 0.1% formic acid in methanol (B) performed as follows: 3% B for 1.5 min, from 3% to14% B in 2.0 min, from 14% to 60% B in 1.5 min, from 60% to 90% B in 0.5 min, 90% B for 1.0 min, and from 90% to 3% B in 0.10 min; conditioning time was 1.9 min.

The eluates were subjected to analysis by a quadrupole-time-of-flight mass spectrometer (Xevo G2 QTOF MS, Waters) equipped with an electrospray ionization (ESI) source. A voltage of 0.5 kV was applied to the electrospray capillary. The source temperature was at 120 °C, and the desolvation temperature was 450 °C. Nitrogen was used as the desolvation gas (650 L/h) and as the cone gas (65 L/h). The total ion current was recorded in positive ionization mode, with a scan range from 140 to 600 m/z. Quantitative analysis was based on extracted ion chromatograms for the following ions (m/z): 341.1501 (for ornithine), 347.1878 (for D6-ornithine), 279.1457 (for arginine), 286.1897 (for D7-arginine), 307.1770 (for ADMA and SDMA), 314.2209 (for D7-ADMA), 263.1090 (for citrulline), 267.1382 (for D4-cytrulline), 150.0919 (for DMA), and 156.1295 (D6-DMA).

#### 4.5.2. Multiplex Analysis

In a subset of 45 ESCC patients, the serum concentration of 48 cytokines, chemokines, and growth factors was quantified using the BioPlex 200 platform (Bio-Rad, Herkules, CA, USA), incorporating Luminex xMAP^®^ technology. This flow cytometry-based method allows for the simultaneous quantification of multiple analytes in real-time. It utilizes magnetic microspheres conjugated with monoclonal antibodies and fluorescent reading. Two Bio-Plex Pro™ Human Cytokine, Chemokine, and Growth Factor Magnetic Bead–Based Assays—Panel I (27-plex) and Panel II (21-plex)—were used. The 27-plex included the following analytes: eotaxin, IL-1β, IL-1ra, IL-2, IL-4, IL-5, IL-6, IL-7, IL-8, IL-9, IL-10, IL-12p70, IL-13, IL-15, IL-17, IFNγ, IP-10, FGF-2, G-CSF, GM-CSF, MCP-1, MIP-1α, MIP-1β, PDGF-BB, RANTES, TNFα, and VEGF-A. The 21-plex included: IL-1α, IL-2Rα, IL-3, IL-12p40, IL-16, IL-18, CTACK, GRO-α, HGF, IFN-α2, LIF, MCP-3, M-CSF, MIF, MIG, β-NGF, SCF, SCGF-β, SDF-1α, TNF-β, and TRAIL. The concentration of IL-1α, IL-12p40, MCP-3, M-CSF, and TNF-β was below the limit of detection in most of the samples, and they were, therefore, excluded from analysis. All analyses were conducted in duplicates and following the manufacturer’s instructions. Standard curves were drawn using 5-PL logistic regression, and the data were analyzed using BioPlex Manager 6.0 software.

#### 4.5.3. Transcriptomic Analysis

Tissue samples (up to 40 mg) were homogenized in a Fastprep 24 Homogenizer (MP Biomedical, OH, USA) using lysis buffer (PureLink™ RNA Mini Kit, Thermo-Fisher Scientific, Waltham, MA, USA) with 2-mercaptoethanol (100:1) (Sigma-Aldrich, St. Luis, MO, USA). Phenol-chloroform extraction was used for RNA isolation and then purified using a PureLink™ RNA Mini Kit (Thermo-Fisher Scientific), including genomic DNA on-column removal by DNase (PureLink™ DNase Set, Thermo-Fisher Scientific) treatment. Isolated RNA was quantified using NanoDrop 2000 (Thermo-Fisher Scientific). Its purity was evaluation using absorbance ratios: 260/280 and 260/230 nm, while its integrity—the Experion platform, incorporating LabChip microfluidic technology, and Experion RNA StdSens analysis kits (BioRad).

Aliquots of RNA corresponding with 1000 ng per reaction mixture (20 µL) were reversely transcribed using a C1000 termocycler (BioRad) and iScript™ cDNA Synthesis Kit (BioRad) following the manufacturer’s protocol.

Quantitative PCR (qPCR) was conducted on a CFX96 Real-Time PCR thermocycler (BioRad) using SsoFast EvaGreen^®^ Supermix (BioRad). The following cycling conditions were applied: 30 s activation at 95 °C, 5 s denaturation at 95 °C, annealing/extension for 5 s at 61 °C, 45 cycles, followed by melting step (60–95 °C with fluorescent reading every 0.5 °C). The reaction mixture consisted of cDNA (2 µL; diluted 1:5), 2×SsoFast EvaGreen^®^ Supermix (10 µL), 10 nM forward and reverse target-specific primers (1 µL of each), and water up to 20 µL. Primer specificity was assured in the melting curve analysis and an electrophoresis in a high-resolution agarose (SeaKem LE agarose from Lonza, Basel, Switzerland) in TBE with SYBR Green (Lonza) detection. Primers were synthesized by Genomed (Warsaw, Poland), and their sequences are presented in Table 12.

Prior analysis, technical replicates were averaged. The geometric mean of all Cq values in a given sample set was obtained and subtracted from the individual sample Cq (ΔCq) then linearized by 2^^ΔCq^ conversion and normalized to *GAPDH*. The obtained values were referred to as a normalized relative quantity (NRQ) [72] and subjected to statistical analysis.

### 4.6. Statistical Analysis

Normality of distribution was tested using a Kolmogorov–Smirnov test and homogeneity of variances using Levene’s test. Data were analyzed using a *t*-test for independent samples, with Welch correction in case of unequal variances, or one-way ANOVA with a Tukey–Kramer posthoc test and presented as means with 95% confidence interval (CI). Transcriptional data were log-transformed and presented as geometric means with 95% CI. Paired data were analyzed using a *t*-test for paired samples. Logistic regression (stepwise model) with variables entered into the model if *p* < 0.05 and removed if *p* > 0.1 was used to select independent predictors of ESCC. The ROC curve analysis was applied to determine diagnostic power. The area under ROC curve (AUC), indicative of overall marker accuracy, as well as marker sensitivity and specificity, corresponding with optimal cut-off value, were calculated. The Fisher’s exact test was applied for frequency analysis. Correlation analysis was conducted using Spearman rank correlation (*ρ*) or Pearson correlation (*r*), depending on the data character. The following rule of thumb was used to interpret the size of the correlation: 0.80 to 1.00 as very strong correlation, 0.60 to 0.80 as moderate correlation, 0.30 to 0.60 as fair correlation, <0.30 as poor correlation [73]. All calculated probabilities were two-tailed. The *p*-values ≤ 0.05 were considered statistically significant. The entire analysis was conducted using MedCalc Statistical Software version 19.2 (MedCalc Software Ltd., Ostend, Belgium; https://www.medcalc.org; 2020).

## Figures and Tables

**Table 1:** Interrelationship between systemic concentrations of L-arginine/NO pathway metabolites in patients with esophageal squamous cell carcinoma (ESCC) or benign conditions of the esophagus.

Metabolite	ADMA	Arg	Cit	DMA	Orn	SDMA
	**Metabolites in ESCC patients**					
ADMA	x	0.43 ^3^	0.25 ^4^	0.32 ^1^	0.33 ^2^	0.44 ^3^
Arg	0.39 ^2^	x	0.40 ^2^	-	-	0.29 ^1^
Cit	0.47 ^3^	0.30 ^1^	x	-	-	-
DMA	0.22 ^4^	-	0.30 ^1^	x	-	0.46 ^3^
Orn	0.28 ^1^	-	0.24 ^4^	-	x	0.35 ^2^
SDMA	0.35 ^2^	-	-	0.45 ^3^	-	x
	**Metabolites in patients with benign diseases**					

Data presented as Pearson correlation coefficient (r). ADMA, asymmetric dimethylarginine; Arg, arginine; Cit, citrulline; DMA, dimethylamine; Orn, ornithine; SDMA, symmetric dimethylarginine; ^1^, *p* < 0.05; ^2^, *p* < 0.01; ^3^, *p* < 0.001; ^4^, 0.05 < *p* < 0.1; -, no significant association or tendency (*p* > 0.1).

**Table 2:** Correlation between systemic concentration of L-arginine/NO pathway metabolites and circulating cytokines, chemokines, and growth factors.

Cytokine	Arg	Cit	Orn	ADMA	SDMA	DMA	A/ADMA	A/C+O
β-NGF	-	-	-	0.31 ^1^	0.42 ^2^	0.32 ^1^	-	-
CTAK	-	-	-	0.33 ^1^	0.48 ^3^	-	-	0.35 ^1^
EOX	-	0.30 ^1^	-	-	-	-	-	-
FGF2	-	−0.50 ^3^	-	-	-	-	-	-
G-CSF	-	−0.45 ^2^	-	-	-	-	-	-
GM-CSF	-	−0.54 ^3^	-	-	0.33 ^1^	-	-	0.28 ^4^
GROα	-	-	-	0.49 ^3^	0.42 ^2^	0.34 ^1^	-	-
HGF	0.42 ^2^	-	-	0.31 ^1^	0.34 ^1^	0.41 ^2^	-	-
IFNα2	-	-	-	0.42 ^2^	0.41 ^2^	0.34 ^1^	-	-
IFNγ	-	−0.57 ^3^	-	-	-	-	-	-
IL-10	-	−0.55 ^3^	-	-	-	-	-	-
IL-12p70	-	−0.33 ^1^	-	-	-	-	−0.27 ^4^	-
IL-13	-	−0.64 ^3^	-	-	0.36 ^1^	0.26 ^4^	-	-
IL-15	-	−0.31 ^1^	-	-	0.33 ^1^	-	-	-
IL-16	-	-	-	-	0.32 ^1^	0.44 ^2^	-	-
IL-17	-	−0.51 ^3^	-	-	0.29 ^4^	-	-	0.41 ^2^
IL-18	0.27 ^4^	-		0.27 ^4^	0.29 ^4^	0.32 ^1^	-	-
IL-1β	-	−0.45 ^2^	-	-	-	-	-	0.32 ^1^
IL-1ra	-	−0.44 ^2^	-	-	-	-	-	-
IL-2Rα	-	-	-	0.40 ^2^	0.26 ^1^	0.38 ^2^	−0.29 ^4^	-
IL-3	-	-	-	0.40 ^2^	0.45 ^2^	0.39 ^3^	-	-
IL-4	-	−0.38 ^2^	-	-	-	-	-	-
IL-5	-	−0.54 ^3^	-	-	-	-	-	-
IL-6	-	−0.35 ^1^	-	-	-	-	-	-
IL-7	-	-	−0.51 ^3^	-	-	-	-	0.39 ^2^
IL-8	-	−0.44 ^2^	-	-	-	-	-	-
IL-9	-	−0.26 ^4^	-	-	0.27 ^4^	-	-	-
IP-10	-	-	-	-	0.45 ^2^	-	-	-
LIF	-	−0.29 ^1^	-	-	0.47 ^2^	0.38 ^2^	-	0.27 ^4^
MCP-1	0.30 ^1^	-	-	-	0.29 ^1^	-	-	-
MIF	-	−0.37 ^1^	0.30 ^1^	-	-	-	−0.32 ^1^	−0.32 ^1^
MIG	-	-	-	0.25 ^4^	0.34 ^1^	-	-	-
MIP-1α	-	−0.33 ^1^	-	-	0.45 ^2^	-	-	-
MIP-1β	-	−0.55 ^3^	-	-	0.31 ^1^	0.31 ^1^	-	-
PDGF-BB	0.32 ^1^	-	-	-	0.26 ^4^	0.27 ^4^	0.33 ^1^	-
SCF	0.30 ^1^	-	-	0.28 ^4^	0.54 ^3^	0.64 ^3^	-	-
SCGF-β	-	-	-	^-^	0.45 ^2^	0.49 ^3^	-	-
SDF-1α	-	-	-	0.35 ^1^	0.37 ^1^	0.31 ^1^	-	-
TNFα	0.36 ^1^	−0.51 ^3^	-	-	0.27 ^4^	-	-	0.31 ^1^
TRAIL	0.47 ^3^	-	-	0.43 ^2^	-	0.48 ^3^	-	0.28 ^4^
VEGF-A	-	−0.26 ^4^	-	-	-	-	−0.35 ^1^	-

Data present Spearman rank correlation coefficients ρ (rho). Only significant correlations or tendencies are included. Statistical significance is indicated by number in superscript: ^1^, *p* < 0.05; ^2^, *p* < 0.01; ^3^, *p* < 0.001; ^4^, tendency 0.05 < *p* < 0.1. Lack of significant correlation or tendency is denoted by “-”. Arg, arginine; Cit, citrulline; Orn, ornithine; ADMA, asymmetric dimethylarginine; SDMA, symmetric dimethylarginine; DMA, dimethylamine; A/ADMA, arginine-to-ADMA ratio; A/C+O, arginine-to-(citrulline+ornithine) ratio; β-NGF, nerve growth factor β; CTAK, C-C motif chemokine ligand 27 (CCL27); EOX, eotaxin 1 (CCL11); FGF2, fibroblast growth factor β; G-CSF, granulocyte colony-stimulating factor; GM-CSF, granulocyte-macrophage colony-stimulating factor; GROa, growth-regulated alpha protein (CXCL1); HGF, hepatocyte growth factor; IFN, interferon; IL, interleukin; IL-1ra, interleukin-1 receptor antagonist; IL-2Rα, interleukin 2 receptor subunit α; IP-10, interferon gamma-induced protein 10 (CXCL10); LIF, leukemia inhibitory factor; MCP-1, monocyte chemoattractant protein 1; MIF, macrophage migration inhibitory factor; MIG, monokine induced by gamma interferon (CXCL9); MIP, macrophage inflammatory protein; PDGF-BB, platelet-derived growth factor BB; SCF, stem cell factor; SCGFβ, stem cell growth factor β; SDF-1α, stromal cell-derived factor 1α; TNFα, tumor necrosis factor α; TRAIL, tumor necrosis factor (TNF)-related apoptosis inducing ligand; VEGF-A, vascular endothelial growth factor A.

**Table 3:** Association between expression ratio (tumor-to-adjacent) of L-arginine/NO pathway enzymes and esophageal squamous cell carcinoma pathology.

Pathology	Expression Ratio (Tumor-to-Adjacent)							
	*ARG1*	*ARG2*	*DDAH1*	*DDAH2*	*NOS2*	*ODC1*	*PRMT1*	*PRMT5*
TNM ^1^	ns	ns	ns	ρ = −0.43, *p* = 0.006	ns	ρ = −0.46, *p* = 0.003	ns	ρ = −0.33, *p* = 0.039
T ^1^	ns	ns	ns	ρ = −0.31, *p* = 0.051	ns	ρ = −0.39, *p* = 0.012	ns	ns
N1 vs. N0 ^2^	0.14 vs. 0.33, *p* = 0.420	0.5 vs. 0.97, *p* = 0.080	0.5 vs. 0.91, *p* = 0.110	0.94 vs. 3.6, *p* = 0.092	4.1 vs. 20.3, *p* = 0.090	3.8 vs. 8.1, *p* = 0.079	1.5 vs. 2.3, *p* = 0.039	1.1 vs. 2.5, *p* = 0.056
M1 vs. M0 ^2^	0.07 vs. 0.24, *p* = 0.465	0.69 vs. 0.68, *p* = 0.987	0.46 vs. 0.7, *p* = 0.474	0.74 vs. 2, *p* = 0.408	21.1 vs. 7.7, *p* = 0.487	1.6 vs. 6.5, *p* = 0.032	2 vs. 1.8, *p* = 0.788	1.3 vs. 1.7, *p* = 0.671

^1^, Data presented as Spearman rank correlation coefficient rho (ρ); ^2^, data presented as mean expression ratio (tumor-to adjacent) in patients with *n* ≥ 1 cancers as compared to patients with N0 cancers or patients with M1 cancers as compared to patients with M0 cancers. TNM, tumor-node-metastasis cancer staging system; T, depth of primary tumor invasion; N, lymph node metastases; M, distant metastases; ARG, arginase; DDAH, dimethylarginine dimethylaminohydrolase; NOS, nitric oxide synthase; ODC, ornithine decarboxylase; PRMT, protein arginine N-methyltransferase; ns, no significant association or tendency (*p* > 0.1).

**Table 4:** Association between relative expression of L-arginine/NO pathway enzymes in tumors and esophageal squamous cell carcinoma pathology.

Pathology	Relative Gene Expression in Tumors (NRQ)							
	*ARG1*	*ARG2*	*DDAH1*	*DDAH2*	*NOS2*	*ODC1*	*PRMT1*	*PRMT5*
TNM ^1^	ns	ns	ns	ns	ns	ρ = −0.28, *p* = 0.082	ns	ns
T ^1^	ns	ns	ns	ns	ns	ns	ns	ns
N1 vs. N0 ^2^	0.39 vs. 0.42, *p* = 0.909	0.74 vs. 0.94, *p* = 0.503	0.63 vs. 1.1, *p* = 0.119	1.1 vs. 1.6, *p* = 0.239	2.3 vs. 3.5, *p* = 0.344	2 vs. 2.7, *p* = 0.468	1.2 vs. 1.6, *p* = 0.220	1.1 vs. 1.4, *p* = 0.383
M1 vs. M0 ^2^	0.28 vs. 0.43, *p* = 0.743	1.1 vs. 0.8, *p* = 0.568	0.95 vs. 0.8, *p* = 0.743	1.2 vs. 1.3, *p* = 0.895	11.6 vs. 2.3, *p* = 0.024	0.76 vs. 2.7, *p* = 0.023	1.7 vs. 1.3, *p* = 0.444	1.6 vs. 1.2, *p* = 0.479

^1^ Data presented as Spearman rank correlation coefficient rho (ρ); ^2^, data presented as mean relative gene expression (NRQ) in tumors in patients with *n* ≥ 1 cancers as compared to patients with N0 cancers or patients with M1 cancers as compared to patients with M0 cancers. TNM, tumor-node-metastasis cancer staging system; T, depth of primary tumor invasion; N, lymph node metastases; M, distant metastases; ARG, arginase; DDAH, dimethylarginine dimethylaminohydrolase; NOS, nitric oxide synthase; ODC, ornithine decarboxylase; PRMT, protein arginine N-methyltransferase; ns, no significant association or tendency (*p* > 0.1).

**Table 5:** Association between relative expression of L-arginine/NO pathway enzymes in noncancerous tumor-adjacent tissue and esophageal squamous cell carcinoma pathology.

Pathology	Relative Gene Expression in Noncancerous Tumor Adjacent Tissue (NRQ)							
	*ARG1*	*ARG2*	*DDAH1*	*DDAH2*	*NOS2*	*ODC1*	*PRMT1*	*PRMT5*
TNM ^1^	ns	ρ = 0.34, *p* = 0.034	ns	ρ = 0.28, *p* = 0.084	ns	ρ = 0.30, *p* = 0.061	ns	ns
T ^1^	ns	ρ = 0.29, *p* = 0.069	ns	ns	ns	ns	ns	ns
N1 vs. N0 ^2^	2.9 vs. 1.3, *p* = 0.300	1.5 vs. 0.97, *p* = 0.143	1.3 vs. 1.2, *p* = 0.797	1.1 vs. 0.44, *p* = 0.238	0.55 vs. 0.18, *p* = 0.145	0.54 vs. 0.33, *p* = 0.185	0.82 vs. 0.69, *p* = 0.442	1 vs. 0.56, *p* = 0.200
M1 vs. M0 ^2^	4 vs. 1.8, *p* = 0.088	1.6 vs. 1.2, *p* = 0.499	2.1 vs. 1.2, *p* = 0.085	1.7 vs. 0.7, *p* = 0.446	0.55 vs. 0.3, *p* = 0.610	0.48 vs. 0.4, *p* = 0.808	0.88 vs. 0.74, *p* = 0.610	1.2 vs. 0.72, *p* = 0.463

^1^, Data presented as Spearman rank correlation coefficient rho (ρ); ^2^, data presented as mean relative gene expression (NRQ) in noncancerous tumor-adjacent tissue in patients with *n* ≥ 1 cancers as compared to patients with N0 cancers or in patients with M1 cancers as compared to patients with M0 cancers. TNM, tumor-node-metastasis cancer staging system; T, depth of primary tumor invasion; N, lymph node metastases; M, distant metastases; ARG, arginase; DDAH, dimethylarginine dimethylaminohydrolase; NOS, nitric oxide synthase; ODC, ornithine decarboxylase; PRMT, protein arginine N-methyltransferase; ns, no significant association or tendency (*p* > 0.1).

**Table 6:** Interrelationship between expression levels of L-arginine/NO pathway enzymes in tumors.

Gene	*ARG1*	*ARG2*	*DDAH1*	*DDAH2*	*NOS2*	*ODC1*	*PRMT1*	*PRMT5*
***ARG1***		0.40 ^1^	0.36 ^1^	0.53 ^3^	-	0.51 ^3^	-	-
***ARG2***			0.43 ^2^	0.46 ^2^	-	0.47 ^2^	0.34 ^1^	0.58 ^3^
***DDAH1***				0.62 ^3^	-	-	0.48 ^2^	0.32 ^1^
***DDAH2***					0.27 ^4^	0.38 ^1^	0.74 ^3^	0.57 ^3^
***NOS2***						-	0.42 ^2^	0.47 ^2^
***ODC1***							-	0.46 ^2^
***PRMT1***								0.63 ^3^

Data presented as Spearman rank correlation coefficient rho (ρ). ARG, arginase; DDAH, dimethylarginine dimethylaminohydrolase; NOS, nitric oxide synthase; ODC, ornithine decarboxylase; PRMT, protein arginine N-methyltransferase; ^1^, *p* < 0.05; ^2^, *p* < 0.01; ^3^, *p* < 0.001; ^4^, 0.05 < *p* < 0.1; -, no significant association or tendency (*p* > 0.1).

**Table 7:** Interrelationship between expression levels of L-arginine/NO pathway enzymes noncancerous tumor-adjacent tissue.

Gene	*ARG1*	*ARG2*	*DDAH1*	*DDAH2*	*NOS2*	*ODC1*	*PRMT1*	*PRMT5*
***ARG1***		0.43 ^2^	0.50 ^3^	-	-	-	-	0.47 ^2^
***ARG2***			0.45 ^2^	0.53 ^3^	-	0.59 ^3^	0.60 ^3^	0.63 ^3^
***DDAH1***				0.51 ^3^	-	-	0.66 ^3^	0.66 ^3^
***DDAH2***					-	0.55 ^3^	0.69 ^3^	0.63 ^3^
***NOS2***						0.55 ^3^	0.33 ^1^	-
***ODC1***							0.50 ^3^	0.36 ^1^
***PRMT1***								0.87 ^3^

Data presented as Spearman rank correlation coefficient rho (ρ). ARG, arginase; DDAH, dimethylarginine dimethylaminohydrolase; NOS, nitric oxide synthase; ODC, ornithine decarboxylase; PRMT, protein arginine N-methyltransferase; ^1^, *p* < 0.05; ^2^, *p* < 0.01; ^3^, *p* < 0.001; -, no significant association or tendency (*p* > 0.1).

**Table 8:** Association between relative expression of L-arginine/NO pathway enzymes in tumors and circulating cytokines, chemokines, and growth factors as well as systemic concentrations of pathway metabolites.

	*ARG1*	*ARG2*	*DDAH1*	*DDAH2*	*NOS2*	*ODC1*	*PRMT1*	*PRMT5*
ADMA	0.29 ^3^	-	-	-	-	-	−0.49 ^2^	-
Arg	0.29 ^3^	-	-	-	-	-	-	-
Cit	-	0.29 ^3^	-	-	-	-	-	-
SDMA	-	0.30 ^3^	-	-	-	-	-	-
Orn	-	-	−0.39 ^1^	-	-	-	-	-
FGF2	-	-	−0.39 ^1^	-	-	-	-	-
HGF	0.40 ^1^	-	-	-	-	-	-	-
G-CSF	-	-	-	-	0.29 ^3^	-	-	-
IL-3	0.29 ^3^	0.30 ^3^	-	-	-	-	-	-
IL-4	-	-	-	-	0.44 ^2^	-	-	-
IL-5	-	-	-	-	0.41 ^1^	-	-	0.29 ^3^
IL-6	-	-	-	-	0.41 ^1^	-	-	0.31 ^3^
IL-7	-	-	-	-	-	-	-	-
IL-8	-	-	-	-	0.31 ^3^	-	-	-
IL-16	-	0.29 ^3^	-	-	-	-	-	-
IL-17	-	−0.29 ^3^	-	-	-	-	-	-
IL-18	0.39 ^1^	-	-	-	-	-	-	-
IFNγ	-	-	-	-	0.31 ^3^	-	-	-
IFNα2	-	-	-	-	-	0.31 ^3^	-	-
MCP-1	-	-	-	-	-	0.35 ^1^	-	-
SDF-1α	-	0.34 ^1^	-	-	-	-	-	-
TRAIL	-	0.32 ^3^	-	-	-	-	-	-
VEGF-A	-	−0.31 ^3^	-	-	-	-	-	-

Data presented as Spearman rank correlation coefficient rho (ρ); ^1^, *p* < 0.05; ^2^, *p* < 0.01; ^3^, 0.05 < *p* < 0.1; -, no significant association or tendency (*p* > 0.1). ARG, arginase; DDAH, dimethylarginine dimethylaminohydrolase; NOS, nitric oxide synthase; ODC, ornithine decarboxylase; PRMT, protein arginine N-methyltransferase; ADMA, asymmetric dimethylarginine; Arg, arginine; Cit, citrulline; SDMA, symmetric dimethylarginine; Orn, ornithine; FGF2, fibroblast growth factor β; G-CSF, granulocyte colony-stimulating factor; HGF, hepatocyte growth factor; IFN, interferon; IL, interleukin; MCP-1, monocyte chemoattractant protein 1; SDF-1α, stromal cell-derived factor 1α; TRAIL, tumor necrosis factor (TNF)-related apoptosis inducing ligand; VEGF-A, vascular endothelial growth factor A.

**Table 9:** Association between relative expression of L-arginine/NO pathway enzymes in noncancerous tumor-adjacent tissue and circulating cytokines, chemokines, and growth factors as well as systemic concentrations of pathway metabolites.

	*ARG1*	*ARG2*	*DDAH1*	*DDAH2*	*NOS2*	*ODC1*	*PRMT1*	*PRMT5*
ADMA	0.29 ^4^	-	-	-	-	-	-	-
FGF2	−0.29 ^4^	-	-	-	-	-	-	-
G-CSF	-	−0.28 ^4^	-	-	-	-	-	-
GM-CSF	−0.29 ^4^	-	-	-	-	-	-	-
GROα	−0.32 ^4^	-	-	-	-	-	-	-
HGF	−0.39 ^1^	-	-	-	-	-	-	-
IL-3	-	-	-	-	0.37 ^1^	0.30 ^4^	-	-
IL-4	-	-	-	-	-	-	0.31 ^4^	0.32 ^4^
IL-5	-	-	-	0.33 ^4^	-	-	0.33 ^4^	0.33 ^1^
IL-6	-	-	-	-	-	-	-	-
IL-7	-	-	-	-	-	-	0.33 ^4^	0.37 ^1^
IL-8	-	-	-	-	-	-	0.30 ^4^	-
IL-9	-	-	-	0.34 ^1^	0.40 ^1^	0.33 ^4^	-	-
IL-13	−0.33 ^4^	-	-	-	-	-	-	-
IL-15	-	-	-	-	0.29 ^4^	-	-	-
IL-16	−0.33 ^4^	-	-	-	-	0.30 ^4^	-	-
IL-18	-	-	-	-	-	0.37 ^1^	-	-
IL-2Rα	−0.35 ^1^	-	-	-	-	0.29 ^4^	-	-
IFNα2	-	-	-	-	-	0.32 ^4^	-	-
IP-10	-	-	-	0.34 ^1^	-	-	-	-
LIF	−0.29 ^4^	-	-	0.45 ^2^	0.38 ^1^	0.53 ^3^	0.28 ^4^	-
MCP-1	-	-	-	-	0.30 ^4^	-	-	-
MIP-1β	-	0.36 ^1^	-	0.30 ^4^	-	0.38 ^1^	0.37 ^1^	0.38 ^1^
RANTES	-	-	−0.30 ^4^	-	0.28 ^4^	0.45 ^2^	-	-
SCGFβ	-	-	-	0.34 ^1^	-	-	0.29 ^4^	-
SDF-1α	-	-	-	-	0.29 ^4^	-	-	-
SCF	-	-	-	-	-	0.41 ^1^	-	-
TNFα	-	-	-	0.28 ^4^	-	-	0.30 ^4^	-
TRAIL	-	-	-	-	-	0.33 ^4^	-	-

Data presented as Spearman rank correlation coefficient rho (ρ); ^1^, *p* < 0.05; ^2^, *p* < 0.01; ^3^, *p* < 0.001; ^4^, 0.05 < *p* < 0.1; -, no significant association or tendency (*p* > 0.1). ARG, arginase; DDAH, dimethylarginine dimethylaminohydrolase; NOS, nitric oxide synthase; ODC, ornithine decarboxylase; PRMT, protein arginine N-methyltransferase; ADMA, asymmetric dimethylarginine; FGF2, fibroblast growth factor β; G-CSF, granulocyte colony-stimulating factor; GM-CSF, granulocyte-macrophage colony-stimulating factor; GROa, growth-regulated alpha protein (CXCL1); HGF, hepatocyte growth factor; IFN, interferon; IL, interleukin; IL-2Rα, interleukin 2 receptor subunit α; IP-10, interferon gamma-induced protein 10 (CXCL10); LIF, leukemia inhibitory factor; MCP-1, monocyte chemoattractant protein 1; MIP, macrophage inflammatory protein; RANTES, regulated on activation, normal T-cell expressed and secreted (CCL5); SCF, stem cell factor; SCGFβ, stem cell growth factor β; SDF-1α, stromal cell-derived factor 1α; TNFα, tumor necrosis factor α; TRAIL, tumor necrosis factor (TNF)-related apoptosis inducing ligand.

**Table 10:** Characteristic of study population: metabolomic cohort.

Parameter	Benign	ESCC	*p*-Value
*N*	62	61	-
Sex (F/M), *n*	26/36	24/37	0.855 ^1^
Age [y], mean ± SD	61.5 ± 10.2	60.4 ± 7.3	0.509 ^2^
TNM (I/II/III/IV), *n*	-	10/18/14/19	-
T (1/2/3/4), *n*	-	10/10/21/20	-
N (0/≥1), *n*	-	25/36	-
M (0/1), *n*	-	42/19	-

*N*, number of patients; F/M, female-to-male ratio; ESCC, esophageal squamous cell carcinoma; yf, years; SD, standard deviation; TNM, tumor-node-metastasis cancer staging system; T, primary tumor extension; N, lymph node metastasis; M, distant metastasis. ^1^ Fisher exact test; ^2^ Welch test.

**Table 11:** Characteristic of study population: transcriptomic cohort.

Parameter	ESCC
*N*	40
Sex (F/M), *n*	15/25
Age [y], mean ± SD	57.9 ± 6.9
Stage (I/II/III/IV)	3/12/19/5
Primary tumor, T (1/2/3/4)	2/11/20/7
Lymph node metastasis, N (no/yes)	19/21
Distant metastasis, M (no/yes)	35/5

*N*, number of patients; F/M, female-to-male ratio; ESCC, esophageal squamous cell carcinoma; y, years; SD, standard deviation; TNM, tumor-node-metastasis cancer staging system; T, depth of tumor invasion; N, lymph node metastasis; M, distant metastasis.

**Table 12:** Primers’ sequences.

Gene	Name	Accession No.	Primer sequence 5′→3′	Size [bp]
*ARG1* ^1^	Arginase-1	NM_001244438.2	F: tcatctgggtggatgctcacac R: gagaatcctggcacatcgggaa	130
*ARG2* ^1^	Arginase-2	NM_001172.4	F: ctggcttgatgaaaaggctctcc R: tgagcgtggattcactatcaggt	119
*NOS2* ^1^	Inducible nitric oxide synthase	NM_000625.4	F: gctctacacctccaatgtgacc R: ctgccgagatttgagcctcatg	136
*ODC1* ^1^	Ornithine decarboxylase	NM_002539.3	F: ccaaagcagtctgtcgtctcag R: cagagattgcctgcacgaaggt	162
*PRMT1* ^1^	Arginine *N*-methyltransferase-1	NM_001536.5	F: tgcggtgaagatcgtcaaagcc R: ggactcgtagaagaggcagtag	142
*PRMT5* ^1^	Arginine *N*-methyltransferase-5	NM_006109.5	F: ctagaccgagtaccagaagagg R: cagcatacagctttatccgccg	136
*DDAH1* ^1^	Dimethylarginine dimethylaminohydrolase-1	NM_012137.4	F: atgcagtctccacagtgccagt R: ttgtcgtagcggtggtcactca	151
*DDAH2* ^1^	Dimethylarginine dimethylaminohydrolase-2	NM_001303007.2	F: ctttcttcgtcctgggttgcct R: ctccagttctgagcaggacaca	136
*GAPDH* ^2^	Glyceraldehyde-3-phosphate dehydrogenase	NM_001256799.3	F: tagattattctctgatttggtcgtattgg R: gctcctggaagatggtgatgg	223

^1^, primer sequences were as proposed by Origene (www.origene.com); ^2^, primers were designed using Beacon Designer Probe/Primer Design Software (BioRad), validated in silico by Blast analysis, and their specificity tested by means of melting curve analysis and an electrophoresis in a high-resolution agarose. Forward and reverse primer sequences are denoted by “F” and “R”, respectively.

## Abbreviations

ADMA	Asymmetric dimethylarginine
Arg	Arginine
ARG	Arginase
ASS1	Argininosuccinate lyase
AUC	Area under receiver operating characteristics (ROC) curve
CAT	Cationic amino acid transporter
Cit	Citrulline
CRC	Colorectal cancer
CTAK	C-C motif chemokine ligand 27 (CCL27)
DDAH	Dimethylarginine dimethylaminohydrolase
DMA	Dimethylamine
EOX	Eotaxin-1
ESCC	Esophageal squamous cell carcinoma
FGF2	Fibroblast growth factor β
G-CSF	Granulocyte colony-stimulating factor
GM-CSF	Granulocyte-macrophage colony-stimulating factor
GROa	Growth-regulated alpha protein (CXCL1)
HGF	Hepatocyte growth factor
IFN	Interferon
IL	Interleukin
IL-1ra	Interleukin-1 receptor antagonist
IL-2Rα	Interleukin 2 receptor subunit α
IP-10	Interferon gamma-induced protein 10 (CXCL10)
LC-MS/MS	Liquid Chromatography with tandem mass spectrometry
LIF	Leukemia inhibitory factor
MCP-1	Monocyte chemoattractant protein 1
MIF	Macrophage migration inhibitory factor
MIG	Monokine induced by gamma interferon (CXCL9)
MIP	Macrophage inflammatory protein
NOS	Nitric oxide synthase
NRQ	Normalized relative quantity
ODC	Ornithine decarboxylase
Orn	Ornithine
PDGF-BB	Platelet-derived growth factor BB
PRMT	Protein arginine N-methyltransferase
RANTES	“Regulated on activation, normal T-cell expressed and secreted” (CCL5)
SCF	Stem cell factor
SCGFβ	Stem cell growth factor β
SDF-1α	Stromal cell-derived factor 1α
SDMA	Symmetric dimethylarginine
TNFα	Tumor necrosis factor α
TNM	Tumor-node-metastasis cancer staging system
TRAIL	Tumor necrosis factor (TNF)-related apoptosis inducing ligand
VEGF-A	Vascular endothelial growth factor A
β-NGF	Nerve growth factor β
